# Virulence of *Mycobacterium avium* Subsp. *hominissuis* Isolated From Feline Thyroid and Pancreatic Tissues in a Murine Infection Model

**DOI:** 10.1155/vmi/7207350

**Published:** 2026-08-21

**Authors:** Adeliane Castro da Costa, Fábio Muniz de Oliveira, Fabrício Camargo, Lázaro Moreira Marques-Neto, Ísis Assis Braga, Danieli Brolo Martins, André Kipnis, Ana Paula Junqueira-Kipnis

**Affiliations:** ^1^ Tropical Pathology and Public Health Institute of the Federal University of Goiás, Goiânia, Goiás, Brazil; ^2^ Immunology Laboratory, Institute of Health Sciences, Federal University of Jataí, Jataí, Goiás, Brazil, nsw.gov.au; ^3^ Clinical Analysis Laboratory, Institute of Agricultural Sciences, Federal University of Jataí, Jataí, Goiás, Brazil; ^4^ Veterinary and Animal Sciences School of the Federal University of Goiás, Goiânia, Goiás, Brazil

**Keywords:** feline, immune response, mononuclear cells, mycobacteriosis

## Abstract

Mycobacteria infection is a serious public health problem and has been described as causing diseases in domestic animals. The objective of this study was to evaluate the immunopathogenesis of *Mycobacterium avium* subspecies *hominissuis* isolated from a domestic cat in mice. Mice were intravenously infected with 10^6^ CFU, and the spleen, lung, liver, and submandibular glands were collected for a histopathology study and bacillary load evaluation. Lung cells, such as macrophages, NK1.1 cells, and CD4^+^ and CD8^+^ T lymphocytes were evaluated by flow cytometry. The infection spread to the liver, lungs, lymph nodes, and submandibular salivary gland, causing severe necrotic lesions and inducing increased levels of lung macrophages, NK, and dendritic cells, as well as specific CD4^+^IFN‐γ^+^ and CD8^+^IFN‐γ^+^ T lymphocytes. Thus, *Mycobacterium avium* subsp*. hominissuis* presented here induced pathogenicity and immune responses in the mouse model, as other mycobacterial infections, indicating that this bacterium could induce serious disease in animals.

## 1. Introduction

*Mycobacterium avium* Complex (MAC) species are nontuberculous bacteria widely disseminated in several animal species around the world and, currently, are subdivided into four genetically similar subspecies: *M. avium* subsp. *hominissuis* (MAH), that infect swine, other animals and can act on opportunistic pathogens in humans; *M. avium* subsp. *paratuberculosis* (MAP) associated with ruminant diseases; *M. avium* subsp*. silvaticum* (MAS), mainly affects wood pigeons; and *M. avium* subsp. *avium* (MAA), the main cause of avian mycobacteriosis [1].

MAH represents an increasing public health concern, as infections in pets have been frequently reported, and the potential transmission of the bacterium from dogs and cats to humans has been considered [2–7]. Studies have shown that MAH infections in pets are often associated with lesions in the inguinal, mediastinal, mesenteric, and prescapular lymph nodes, characterized by numerous macrophages and neutrophils, as well as granulomatous inflammation in the liver, spleen, and tonsils with typical caseous necrosis. Moreover, mycobacteria have been detected in histiocytes, lungs, skin, intestine, and bone marrow and have also been associated with pyogranulomatous meningoencephalitis in cats, indicating a systemic pattern of infection [3, 8–13].

Domestic animals exhibiting systemic mycobacteriosis have often been associated with zoonoses, causing serious public health problems [9–13]. The understanding of the immunopathological aspects generated after the infection by *M. avium* and other nontuberculosis mycobacteria may be essential for the development of new diagnostic and treatment techniques against those bacteria. In this sense, the objective of this study was to analyze the host–pathogen interactions of an isolate of MAH obtained from an infected cat, using the mouse model of infection. Although a recent work used MAH‐infected mouse as a model to test new drugs effectiveness [14], this is the first work, to our knowledge, to evaluate the innate and specific immune responses using mice.

To investigate host–pathogen interactions and their consequences during infection, we evaluated tissue lesion development and bacterial load in different organs using a mouse model of infection.

## 2. Materials and Methods

### 2.1. Isolation and Identification of Mycobacterium Infection

A three‐year‐old spayed female Persian cat, housed exclusively indoors, was attended to the Veterinary Hospital of the Federal University of Goiás (HV‐UFG). The cat had been diagnosed with feline atopic dermatitis (FAD) and was referred for further evaluation due to progressive weight loss, apathy, pruritus, and recurrent lesions in the cervical region, and the medical history included previous treatments with cefovecin, itraconazole, cyclosporine, and oclacitinib. On physical examination, thyroid and pancreas enlargement were noted. Ultrasonography of the thyroid and abdomen confirmed the increased volume of the thyroid and revealed splenomegaly, as well as pancreatic enlargement with small nodular areas.

The animal was submitted to fine‐needle aspiration cytology (FNAC) of thyroid and pancreas nodules for mycobacterial infection investigation. The smear was stained by the Fite–Faraco method and showed the presence of acid‐fast bacilli (AFB). Thus, the material was cultivated in Lowenstein Jensen (LJ) medium with or without pyruvate. Around 3 weeks after inoculum, bacterial growth was observed in both culture media where samples were inoculated. An isolated colony was inoculated into Middlebrook 7H9 liquid medium at 37°C for 14 days, and after that purity of culture was confirmed by microscopic analyses after Ziehl–Neelsen staining. Genomic DNA was extracted by using the AxyPrep bacterial genomic DNA miniprep kit according to the instructions of the manufacturer (Axygen‐Biosciences).

### 2.2. Amplification and Partial Sequencing of rpoB Gene

Identification of genus *Mycobacterium* was done by multiplex real‐time PCR, as described in previous research [15]. Species‐level identification was done by the amplification and sequencing of a 723‐bp fragment of the rpoB gene. The primers MycoF (5′‐GGCAAGGTCACCCCGAAGGG‐3′) and MycoR (5′‐ AGCGGCTGCTGGGTGATCATC‐3′) were used [16]. The PCR mixtures consisted of 1–5 µg of purified genomic DNA, 1 µM of each primer, 250 µM each deoxynucleotide triphosphate, buffer GoTaq (Promega), and 1U of *Taq* DNA polymerase (Invitrogen). The PCR reaction was carried out in the Thermocycler Biocycler with a temperature profile of 94°C for 4 min, followed by 35 cycles at 92°C for 45 sec, 64°C for 30 sec, and 72°C for 60 sec and ended with a final elongation step of 72°C for 10 min. The PCR product was purified with a Qiaquick PCR purification kit (QIAGEN) and then sequenced on both DNA strands. The obtained *rpoB* sequences were analyzed and combined into a single consensus sequence using SnapGene (GLS Biotech). To identify species of the isolate, the consensus sequence was submitted to the BLAST tool from NCBI and compared with reference sequences available in the NCBI GenBank database using BLASTn to determine species identity. To draw the phylogenetic tree, the partial rpoB sequence representative *Mycobacterium* species were obtained from NCBI using the primer designing tool applying the sequences of primers used in the PCR, including MAP (NC_002944.2), MAH (CP029332.1), MAA (GQ153306.1), *Mycobacterium kansasii* (NC_022663.1), *Mycobacterium chelonae* (NZ_CP007220.1), *Mycobacterium abscessus* (NC_010397.1), *Mycobacterium fortuitum* (AY147172.1), *Mycobacterium mucogenicum* (AY147171.1), *Mycobacterium smegmatis* (NC_008596.1), *Mycobacterium bovis* (NC_002945.4), *Mycobacterium tuberculosis* H37Rv (NC_000962.3), and *Mycobacterium leprae* (AL583923.1). Pairwise sequence comparison for nucleic acid sequence homology and drawing phylogenetic tree were performed using the ClustalX program (Version 2.1), and phylogenetic relationships were inferred using the neighbor‐joining method with 1000 bootstrap replicates, and evolutionary distances were calculated using the Kimura two‐parameter model. The resulting phylogenetic tree was visualized and edited using FigTree (Version 1.4.2).

### 2.3. Animal Experimentation

Four‐week‐old isogenic C57BL/6 mice from the Tropical Pathology and Public Health Institute of the UFG were used. The animals were handled according to the guidelines of the Conselho Nacional de Experimentação Animal (CONCEA). The study was approved by the ethics committee for animal use of the UFG, under protocol number 060/17.

### 2.4. MAH_01 Infection of C57BL/6 Mice

The MAH culture at logarithmic growth was harvested at 6000*x* g for 5 min, and the culture pellet was resuspended in PBS containing 0.05% Tween 80. After that, the inoculum concentration was adjusted for an optic density (OD) corresponding to 107 CFU/mL. Subsequently, 100 µL of the bacterial suspension was inoculated into the tail vein of ten mice, while 100 µL of saline solution was administered to nine mice, which served as the control group. One day postinfection (dpi), one mouse was euthanized, and the bacterial burden was confirmed by plating lung homogenates on Middlebrook 7H11 agar medium supplemented with OADC [17]. Groups of three infected and three control animals were euthanized at 30 and 60 dpi for bacterial load determination and histopathological analysis.

### 2.5. Evaluation of Bacillary Load

The right anterior (mediastinal) lung lobe, liver, and spleen from infected and control groups were homogenized and plated under the same conditions described above. The bacterial load was determined after 21 days of incubation at 37°C with 0.05% CO_2_ [17].

### 2.6. Histopathology

The lungs were perfused with 0.05% PBS by injection into the right ventricle. The posterior right lobe was collected and fixed with 10% buffered formaldehyde. In addition, the right lobe of the liver, the parathyroid lymph nodes, and the submandibular salivary glands were obtained. The collected organs were conditioned in cassettes on white paper and fixed in 10% buffered formalin for histopathological analysis. The samples were sectioned in 5 μm slides and stained with hematoxylin and eosin (H&E) for microscopic analyses (Axio scope. A1‐ Carl Zeiss).

### 2.7. Flow Cytometry

The left lung lobes were collected at 30 and 60 dpi and processed as previously described by Da Costa et al. [17]. After collection, the lungs were treated with a solution of DNAse IV (30 μg/mL; Sigma‐Aldrich) and Collagenase III (0.7 mg/mL; Sigma‐Aldrich) for 1 h at 37°C. To obtain the cell suspension, the tissues were strained on a 70‐μm filter (BD Biosciences, Lincoln Park, NJ). The erythrocytes were lysed with lysis solution (0.15 M NH4CL, 10 mM KHCO3), then the cells were washed and resuspended in cRPMI medium and adjusted to 1 x 10^6^ cells/mL.

Cells were labeled with anti‐CD86 (BD; clone 16‐10A1)/anti‐CD4 (BD; clone RM4‐5)/anti‐CD3/(BD; clone 145‐2c11) (FITC), anti‐CD11c (BD; clone N418)/anti‐CD8 (BD; clone 53–6.7) (PE) and anti‐CD11b (BD; clone M1/70) (PercP), anti‐NK1.1 (BD; clone PK136)/anti‐F4/80 (Biolegend; clone BM8), and the cells were fixed with PERM FIX (BD Cytofix/CytopermTM) for 20 min at 2°C–8°C, after this period, the cells were permeabilized with PERMWASH (BD Cytofix/CytopermTM) for 20 min at 2°C–8°C followed by anti‐IFN‐γ (BD; clone XMG1.2) (APC) incubation for another 20 min. Then, 200 μL of PBS azide were added, and after further centrifugation, the cells were resuspended in 200 μL of PBS azide, and the acquisition was performed at FACS verse.

To evaluate the alveolar macrophage population, first, the events were gated at monocyte size and granularity and after gating the F4/80+ population, two subpopulations were evaluated: The F4/80^+^CD11c^+^CD11b low were considered dendritic cell population, and the F4/80^+^CD11c^+^CD11b high were considered the total macrophage population. The median fluorescence intensity (MFI) of the CD86 molecule, a key costimulatory molecule involved in the induction of specific immune responses, was assessed in all cell populations. To investigate the NK1.1^+^ population, the gating strategy was to gate lymphocytes by size and granularity, then selected the CD8‐negative population, followed by the quadrant settings using NK1.1 and CD3. The NK1.1 cell population was defined as CD8‐CD3‐NK1.1+. These expressions of CD11b using the MFI were used to evaluate the activating status.

The evaluation of the specific immune response was performed by analyzing IFN‐γ–producing CD4^+^ and CD8^+^ T lymphocyte populations. Lung cells from control and infected mice were processed and stimulated with the ESAT‐6 protein from *M. tuberculosis* to assess the induction of IFN‐γ production by CD4^+^ and CD8^+^ lymphocytes. Lymphocytes were gated based on size and granularity and then classified as CD4+ or CD8+ subpopulations. Then, these events (cells) were evaluated using quadrant settings for IFN gamma positivity. All cytometric results involving specific immune responses used the uninfected mice (control) to determine the negative threshold.

### 2.8. Statistical Analyses

Data were tabulated on Microsoft Office Excel 2011 and analyzed by Prism software (Version 5.0c, GraphPad). The obtained results were evaluated for normality using the Shapiro–Wilk test. One‐way ANOVA followed by Tukey’s multiple comparisons were used for CFU results, and for the other results, two‐way ANOVA followed Bonferroni’s multiple comparison test. All results were considered parametric; therefore, the results are represented by the mean and standard deviation of each group and experimental point. The results of each experimental point were compared with their respective controls. A confidence interval of 95% was considered for all experiments. All experiments were repeated three times, the results being reproducible. (Version 5.0c, GraphPad).

## 3. Results

### *3.1.* MAH

**FIGURE 1:**
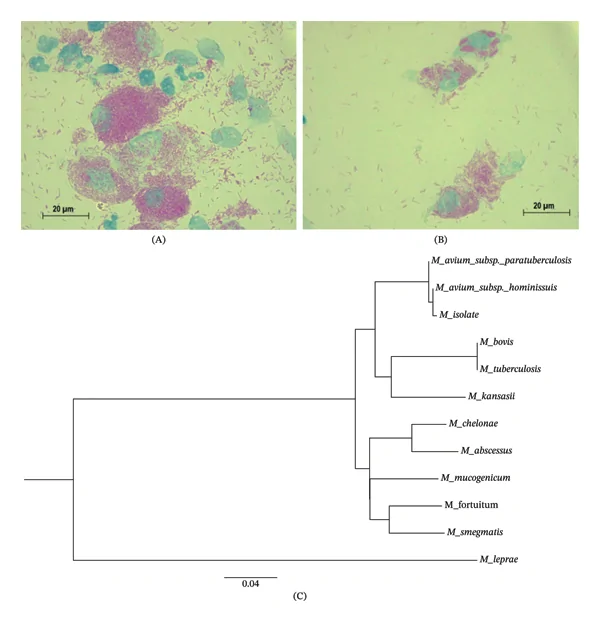
Cytologic findings and partial *rpoB* gene phylogeny from feline thyroid and pancreatic tissues obtained by fine‐needle aspiration. Acid‐fast, rod‐shaped bacilli predominantly located inside macrophages in fine‐needle aspirates from the thyroid (A) and pancreas (B) stained by the modified Fite–Faraco technique. (C) Phylogenetic tree based on partial *rpoB* gene sequence alignment between the isolate and related *Mycobacterium* species. See Methods section for accession numbers.

Isolation results showed the presence of multiple AFB within the macrophages, both in thyroid and pancreas samples. Partial sequencing of the *rpoB* gene revealed that the isolated strain shared high similarity with members of the MAC and was most closely related to MAH, designated as the MAH_01 isolate (Figure 1).

### 3.2. MAH_01 Induces Multiorgan Lesions With Hepatic Tropism

**FIGURE 2:**
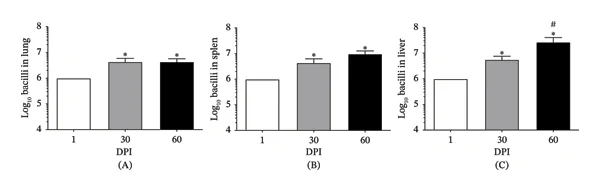
Bacterial load in the lungs, spleen, and liver of C57BL/6 mice infected with MAH_01. Bacterial load (CFU) measured at 1, 30, and 60 days postinfection (dpi) in the lung lobes (A), liver (B), and spleen (C). ^∗^ *p* < 0.05 indicates a significant difference compared to Day 1. ^#^ *p*  < 0.05 indicates a significant difference between Day 60 and Day 30.

Infected mice exhibited an increased bacillary load in the lungs, spleen, and liver compared to Day 1. Notably, the liver of infected animals showed a steady increase in bacillary load up to 60 dpi, compared to Day 30 (#, *p* < 0.05; Figure 2).

**FIGURE 3:**
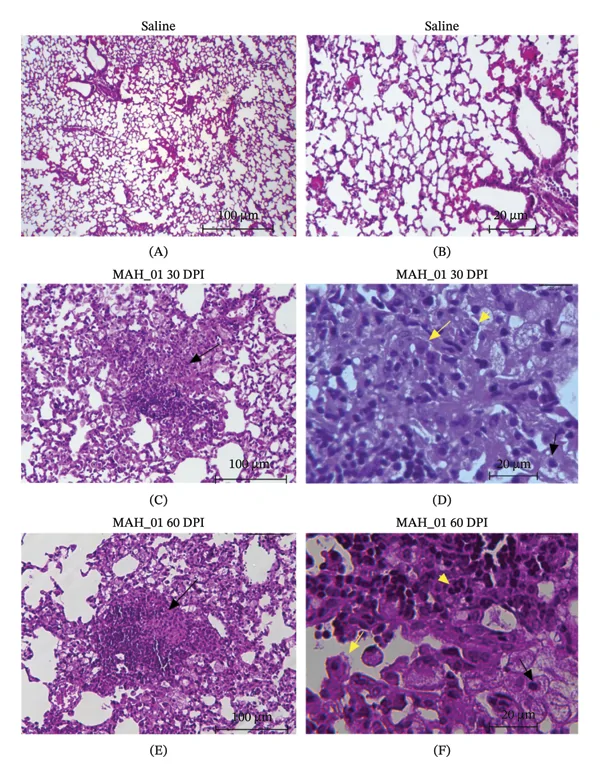
Histologic cross sections of the lung in C57BL/6 mice infected with MAH_01 at 30‐ and 60‐day postinfection (dpi). (A, B) Noninfected control. (C, E) Infiltrates mainly consisting of mononuclear cells, H&E, bar scale = 100 μm. (D, F) Mononuclear cells (yellow arrows); polymorphonuclear cells (black arrowhead); foamy macrophages (black arrows), bar scale = 20 μm.

Histopathological sections of lungs from mice infected with MAH_01 were evaluated at 30 and 60 dpi showing inflammatory infiltrates predominantly composed of mononuclear cells, polymorphonuclear cells and foamy macrophages, while preserving the overall tissue architecture. In addition, numerous foci of bronchial epithelial hyperplasia, associated with fibroblast proliferation and collagen deposition, were observed (Figure 3).

**FIGURE 4:**
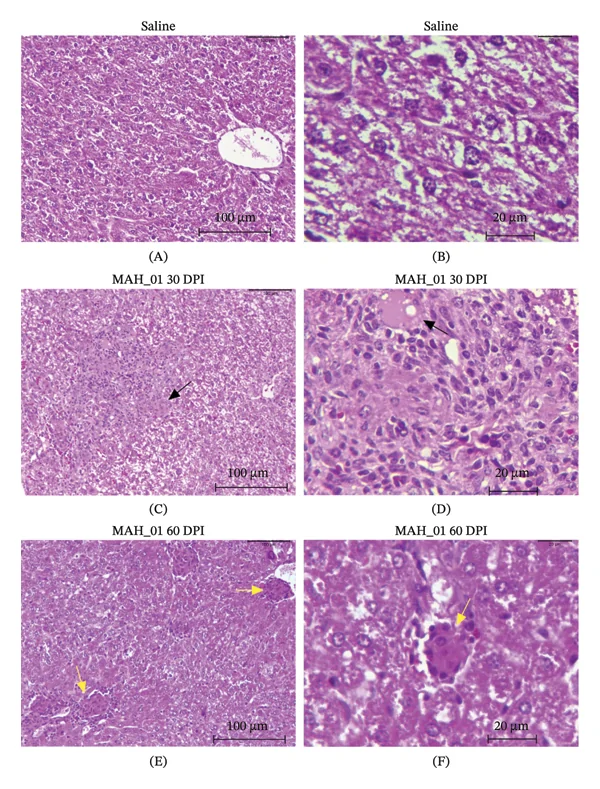
Histologic cross sections of the liver in C57BL/6 mice infected with MAH_01 at 30‐ and 60‐day postinfection (dpi). (A, B) Noninfected control, H&E, bar scale = 100 μm and 20 μm. (C, D) Diffuse and severe hepatocellular necrosis (arrows), H&E, bar scale = 100 μm and 20 μm. (E, F) Multifocal reactive structures that will give rise to granulomas (yellow arrows), H&E, bar scale = 100 μm and 20 μm.

In liver sections at 30 and 60 dpi, extensive inflammatory lesions were observed, characterized by discrete structures resembling granulomas. These consisted of mononuclear cells at various stages of cell death (i.e., pyknosis, karyorrhexis, and karyolysis), with cytoplasmic hydropic changes and the onset of caseous necrosis. Multiple multinucleated giant cells, along with epithelioid cells, were also observed. In addition, several hemorrhagic foci were noted, along with multiple necrotic lesions exhibiting a “dry lake bed” appearance, leading to disruption of tissue architecture. Cells undergoing cell death were observed throughout the liver (Figure 4).

**FIGURE 5:**
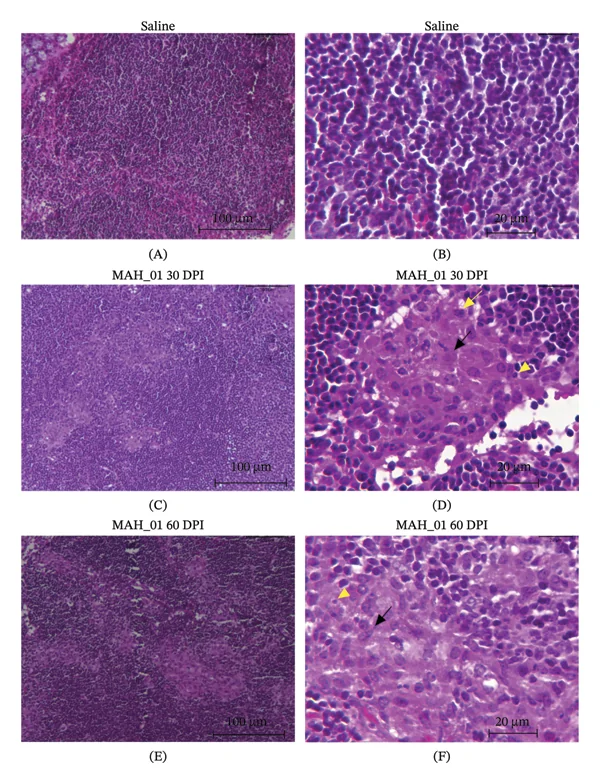
Histologic cross sections of lymph node in C57BL/6 mice infected with MAH_01 at 30‐ and 60‐day postinfection (dpi). (A, B) Noninfected control, H&E, bar scale = 100 μm and 20 μm. (C, E) Diffuse necrosis, H&E, bar scale = 100 μm and 20 μm. (D, F) Pyknosis (yellow arrow heads); karyorrhexis (yellow arrows); karyolysis (black arrows), H&E, bar scale = 100 μm and 20 μm.

**FIGURE 6:**
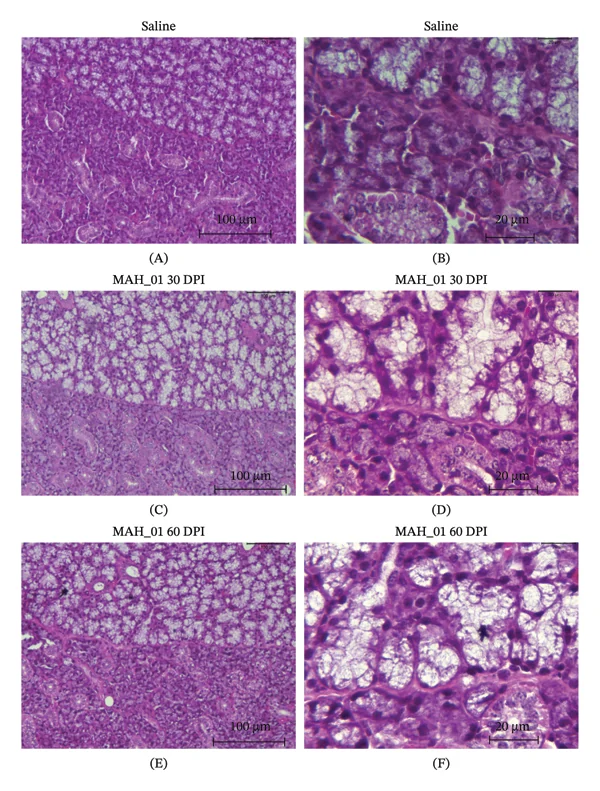
Histologic cross sections of the salivary gland in C57BL/6 mice infected with MAH_01 at 30‐ and 60‐day postinfection (dpi). (A, B) Noninfected control, H&E, bar scale = 100 μm and 20 μm. (C–F) Hypertrophy of the salivary gland, H&E, bar scale = 100 μm and 20 μm.

The overall lymph node architecture was preserved; however, a reduction in cellularity was observed at 30 dpi, which recovered by 60 dpi. Several scattered inflammatory lesions with early necrosis were present. In addition, numerous cells exhibited features of cell death, including pyknosis, karyorrhexis, and karyolysis throughout the lymph nodes (Figure 5). Hypertrophy of the submandibular salivary gland was observed at 30 dpi, which slightly decreased by 60 dpi (Figure 6).

### 3.3. Lung Immune Cell Recruitment Following MAH_01 Infection

**FIGURE 7:**
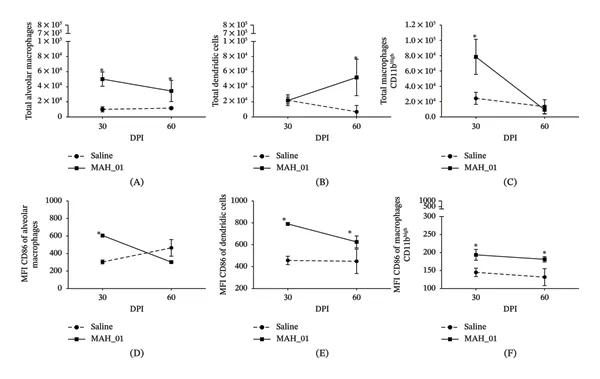
Flow cytometry analysis of lung macrophage and dendritic cell recruitment in MAH_01‐infected mice at 30‐ and 60‐day postinfection (dpi). Absolute numbers of alveolar macrophages F4/80^+^CD11c^+^CD11b^low^ (A). Absolute numbers of dendritic cells F4/80^+^CD11c^+^CD11b^high^ (B). Absolute numbers of macrophages F4/80^+^CD11c^-^CD11b^high^ (C). CD86 median fluorescence intensity (MFI) of alveolar macrophages F4/80^+^CD11c^+^CD11b^low^ (D). CD86 MFI of dendritic cells F4/80^+^CD11c^+^CD11b^high^ (E). CD86 MFI of macrophages F4/80^+^CD11c^-^CD11b^high^ (F). ^∗^ *p* < 0.05 significant differences between MAH_01‐infected (closed squares) and control (closed circles) groups at 30‐ and 60‐dpi.

MAH_01 infection in mice induced an increase in the alveolar macrophage population at 30 dpi compared to the control group (30dpi: *p* < 0.001; control: 10067 ± 3411; MAH_01: 50133 ± 9407). However, the expression of the CD86 molecule was increased in alveolar macrophages only at 30 dpi (*p* < 0.00001); MFI control: mean = 304 ± 21,9; MAH_01 mean: 605 ± 15). Moreover, MAH_01 infection promoted increased migration of DCs cells at 60 dpi (60dpi: *p* < 0.001; control: 6866 ± 8406; MAH_01: 52366 ± 24119). Nevertheless, DCs exhibited higher CD86 expression at both 30 and 60 dpi compared to DCs from control animals (*p* < 0.05); MFI: control/30dpi: 457 ± 38; MAH_01/30dpi: 790 ± 8,7; *p* < 0.05: control/60dpi: 448 ± 112; MAH_01/60dpi: 623 ± 53). Similarly, MAH_01 infection increased the population of migrating blood macrophages only at 30 dpi (30dpi: *p* < 0.01; control: 24300 ± 7961; MAH_01: 78500 ± 22952). Regardless, these macrophages expressed higher levels of CD86 at both 30 and 60 dpi (*p* < 0.01; MFI: control/30dpi: 145 ± 11; MAH_01/30dpi: 195 ± 15; *p* < 0.01: control/60dpi: 131 ± 23; MAH_01/60dpi: 181 ± 7,5) (Figure 7).

**FIGURE 8:**
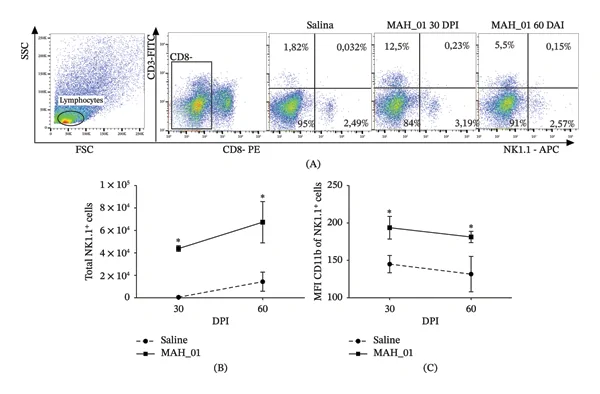
Flow cytometry analysis of the lung NK1.1 cell in MAH_01–infected mice at 30‐ and 60‐days postinfection (dpi). Gating strategy: The lymphocytes were gated by size and granularity, then the CD8‐cells were gated. CD8‐ were evaluated using quadrant settings comparing CD3 and NK1.1 positive cells. NK1.1 cells were defined as CD8‐CD3‐NK1.1+ (A). Absolute numbers of NK1.1+ cells at 30‐ and 60‐dpi (B). Median fluorescence intensity (MFI) of CD11b by NK1.1+ at 30‐ and 60‐dpi (C). ^∗^Significant differences between MAH_01‐infected (closed squares) and control (closed circles) groups (*p* < 0.05).

MAH_01 induced an increased level of NK1.1^+^ cells 30 and 60 dpi compared to the control group (30dpi: *p* < 0.0001, control: 565,3 ± 10,69; MAH_01: 43833 ± 264; 60dpi: *p* < 0.05; control: 14373 ± 8486; MAH_01: 67367 ± 18407). MFI of CD11b was greater among NK cells from MAH_01‐infected mice compared to the control group at all evaluated periods (30dpi: *p* < 0.05; MFI control: 145 ± 11,53; MAH_01: 193,7 ± 15,14; 60dpi: *p* = 0.0255; MFI control: 131,7 ± 23,59; MAH_01: 181,3 ± 7,5) (Figure 8).

**FIGURE 9:**
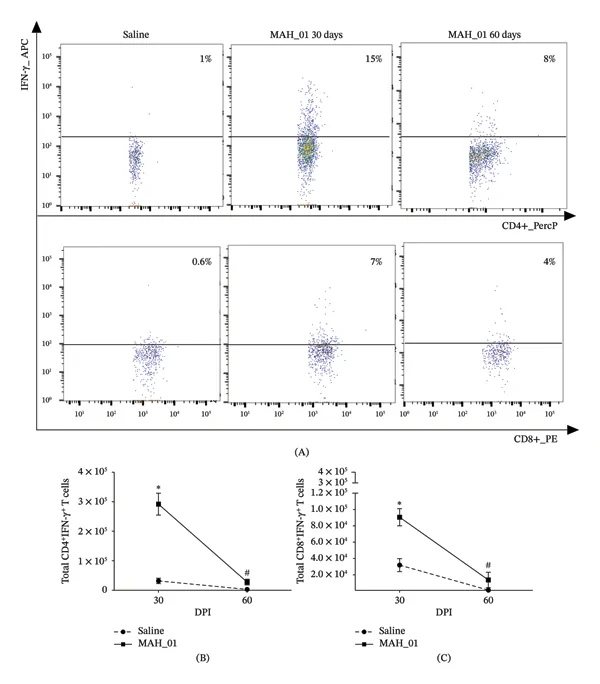
Flow cytometry analysis of the lung CD4^+^IFN‐γ^+^ and CD8^+^IFN‐γ^+^ T lymphocytes in C57BL/6 mice at 30‐ and 60‐day postinfection (dpi) with MAH_01. Gating strategy: The lymphocytes were gated by size and granularity, then the CD4 and CD4 positive cells were gated and evaluated using quadrant settings for IFN‐γ. The graphs show representative plots ofCD4^+^/CD8^+^ IFN‐γ + T lymphocytes in lungs (C57BL/6) at 30‐ and 60‐dpi compared to controls animals (A). Absolute numbers of lung CD4^+^IFN‐γ^+^ T lymphocytes at 30‐ and 60‐dpi (B). Absolute numbers of lung CD8+IFN‐γ+ lymphocytes at 30‐ and 60‐dpi (C). ^∗^Significant differences between MAH_01‐infected (closed squares) and control (closed circles) groups (*p* < 0.05). ^#^Significant differences between MAH_01‐infected 30‐ and 60‐dpi.

MAH_01 infection induced a pulmonary influx of CD4^+^IFN‐γ^+^lymphocytes at 30‐dpi compared to the control group (30dpi: *p* < 0.001; control: 31967 ± 9016; MAH_01: 291667 ± 37314). A reduction in CD4^+^IFN‐γ^+^ lymphocytes was observed at 60‐dpi when compared to 30‐dpi (*p* < 0.001; MAH_01 30dpi: 291667 ± 37314; MAH_01 60 dpi: 27867 ± 9613). A similar pattern was observed for CD8^+^IFN‐γ^+^lymphocyte (30dpi: *p* < 0.0001; control:32000 ± 7979; MAH_01: 90600 ± 10553; #: 30‐ vs. 60‐dpi: *p* < 0.0001; MAH_01 30dpi: 90600 ± 10553; MAH_01 60‐dpi: 13760 ± 9488) (Figure 9).

## 4. Discussion

*Mycobacterium avium* infections in cats have been increasingly reported, with bacteria detected in multiple organs, including the lungs, liver, kidneys, lymph nodes, spleen, and brain, as well as in histiocytes of facial muscle tissue and macrophages within abdominal effusion [8, 18–21]. Here, we present some virulence aspects of a MAH (MAH_01) isolated from a cat infection. The species identification was confirmed by the widely accepted and used rpoB gene sequencing technique [16]. Although additional molecular techniques such as IS1245 and IS901 sequencing can be used for further discrimination, the identification of the isolate as MAH was supported by its clustering with reference MAH sequence in the phylogenetic tree presented in Figure 1. Isolation of this mycobacterium from thyroid and pancreatic tissues is rarely reported [19], and to our knowledge, this represents the first report of MAH isolation from these tissues in feline, further supporting its multisystemic nature. The systemic MAH_01 infection observed in this case may be associated with immunosuppression resulting from prior treatment with cyclosporine, as disseminated mycobacteriosis can be linked to the use of immunosuppressive drugs, as well as infections caused by feline leukemia virus, feline immunodeficiency virus, toxoplasmosis, or idiopathic CD4^+^ T‐lymphopenia [20–27].

However, nontuberculous mycobacteria are also capable of causing disease in immunocompetent animals and humans by subverting the host immune response, as observed following MAH_01 inoculation in healthy mice, which exhibited an increase in bacterial load in the liver, lungs, and spleen from Day 1 to Day 60 postinfection, with the highest bacterial proliferation observed in the liver (Figure 2). The pattern of hepatic bacterial load appears to be lower compared to the lung and spleen in experimental infections with *M. avium* and *M. tuberculosis* in murine models. Moreover, the bacterial load tends to stabilize and decline after 6–8 weeks of infection [28, 29].

The high and persistent bacterial load within hepatocytes explains the diffuse and severe hepatocellular necrosis, as well as the multifocal reactive structures observed in the early stages of granuloma formation in the histopathological analysis of MAH‐01–infected animals. The cell death processes detected in the liver, lungs (Figure 3 and 4), and lymph nodes (Figure 5), along with the accumulation of phagocytic cells, are characteristic lesions of the chronic inflammatory response typical of mycobacterioses [28]. In *M. avium* infection, macrophages secrete an apoptosis‐inhibitory protein that promotes the accumulation of lipid droplets and the formation of foamy macrophages, which in turn facilitate bacterial persistence [30].

Previous studies have demonstrated that MAP can establish infection in bovine salivary glands based on transcriptomic profiling analyses conducted in affected animals [31]. Additionally, evidence from a clinical investigation involving four children who exhibited specific reactivity to novel tuberculin’s revealed two cases associated with *M. malmoense* infection and two others reacting to tuberculins of *M. scrofulaceum* and *M. avium-intracellulare*. A histopathological examination of these patients identified granulomatous lesions forming masses in the parathyroid and salivary glands, confirming the mycobacterial presence [32]. Collectively, these observations support the hypothesis that the alterations reported in the present study—such as the hyperplasia of submandibular glands (Figure 6C–F)—may also reflect salivary gland involvement by mycobacteria.

Macrophages and their derivatives have been directly associated with the control and maintenance of several mycobacteria infections and may contribute to the immunopathology of the bacillus. The gradual increase in the alveolar macrophage population observed in the lungs of *MAH_01*‐infected mice, together with the downregulation of CD86 molecule expression up to 60‐dpi (Figure 7), suggests a shift toward a regulatory or foamy macrophage phenotype, and this modulation of CD86 expression reflects the pathogen’s ability to subvert host immune activation and establish a long‐term infection within the lungs [18, 30].

In contrast, the dendritic cell population increased only at 60‐dpi, although these cells exhibited higher CD86 expression at both 30‐ and 60‐dpi compared to the control group (Figure 7B and E). Even though activation in myeloid subsets was inferred from the upregulation of CD86 as functional outputs, other molecules involved in activation were not evaluated (such as: MHC‐II or cytokines). Surprisingly, CD11b ^high^ macrophages increased only at 30‐dpi (Figure 7C); however, they expressed the CD86 molecule at both 30‐ and 60‐dpi (Figure 7F). Our results agree with the studies showing that *in vitro* MAH‐infected dendritic cells increase CD80 and CD86 expression, as reported by Kim et al. [33].

Once NK cells were directly correlated with mycobacteria infection [21], it was questioned whether this *Mycobacterium* strain could generate NK cell activation. Analysis of NK cells (Figure 8A) shows that MAH_01 induced an increase in the NK1.1^+^ cell population after 30‐ and 60‐dpi (Figure 8B), and that these cells express more CD11b at both 30‐ and 60‐dpi, as compared with the control group (Figure 8C).

*M. avium* causes a pattern of granulomatous lesions very close to the tubercular lesions caused by *M. tuberculosis* and *M. bovis*, with delayed‐type hypersensitivity response, characterized by granulomatous inflammation, and the progression of the disease also depends on the ability of the macrophages to inhibit intracellular growth of the organisms [34–36]. In our model, the MAH_01 was not controlled by the animals’ immune response even with the development of NK cells population which can be the source of IFN‐γ during the onset of infection [37], and the innate immunity seems to be diminished after 60 days, with decreasing in activation marker (CD86) in alveolar macrophage as well as diminishing in the CD11b^high^ population which are potential effectors of acute and chronic lung inflammation [38].

It has been demonstrated that *M. avium* produces some proteins of the ESAT‐6 family, which can be found in the cell wall of this bacterium [22]. Since ESAT‐6 of *M. tuberculosis* has been directly involved with the induction of T lymphocytes in the murine model [23], it has been proposed that MAH_01 can modulate the cellular immune response during the infection in C57BL/6 mice. Because of the similarity between ESAT‐6 from Mtb and proteins found in the *M. avium complex*, the lung cells of the mice infected with MAH_01 were processed and stimulated with ESAT‐6 protein from *M. tuberculosis* to verify the induction of IFN‐γ producing by CD4+ and CD8+ lymphocytes.

Previously, it was shown that MAH isolate induced IL‐17, IL‐10, and IFN‐γ after *in vitro* infection of rat splenocytes [10]. Based on that, it was hypothesized that the MAH_01 strain could influence the TCD4 and TCD8 producers of IFN‐γ^+^ differently. It was possible to observe that MAH_01 induced an increase of CD4^+^ IFN‐γ^+^ lymphocytes at 30‐dpi (Figure 9B). Although this induction remained present after 60 days of infection, it is possible to observe a reduction in the number of TCD4^+^IFN‐γ^+^ lymphocytes in comparison to 30 days (Figure 9B). The same can be observed for the CD8^+^IFN‐γ^+^ lymphocytes (Figure 9C). There are several descriptions in the literature that lipids and lipoproteins on the walls of mycobacteria can suppress and control the host’s immune response. In tuberculosis infection, this capacity is used by the bacteria to evade the immune response and to control the active and latent infection phase transition [38, 39].

## 5. Conclusions

We demonstrated by the phylogenetic analysis made with partial sequences of the *rpoB* gene that the *Mycobacterium* responsible for infection in the cat belonged to the *M. avium* species complex and seemed to be closely related to MAH. The infection of C57BL/6 mice with this strain allowed us to observe the establishment of the infection in different organs of the animals. We observed expansion of NK1.1 cells with higher CD11b expression, activated dendritic cells, and increased CD4^+^IFN‐γ^+^ and TCD8^+^ IFN‐γ^+^ lymphocytes. Although the histology demonstrates the presence of lesions in the lungs, liver, lymph nodes, and submandibular salivary gland, the bacterium preferably infects the liver, even after 60 days of infection.

Finally, MAH is a *Mycobacterium* that can cause infection and severe lesions in different organs, inducing innate and specific immune responses with a bacterium that seems to dump the immune response while the bacillary load is still growing. This showed an acute, more pathogenic infection, and probably a pathogen not well evolved to infect the hosts and should be considered as a potential lethal zoonotic agent.

## Author Contributions

Experimental design and setup: A.P.J.‐K., A.K., and D.B.M. Experimental development and data analysis: A.P.J.‐K., A.K., D.B.C., A.C.C., F.M.O., and F.C. Grant PIs that afforded reagents, materials, and analysis tools for all experiments: A.P.J.‐K. and A.K. Critical discussion and writing of the manuscript: A.P.J.‐K., A.K., D.B.M., A.C.C., F.M.O., I.A.B., F.C., L.M.M.‐N., and I.A.B. Cytometry experiments and analysis: A.C.C.

## Funding

This study was financed by the National Council for Scientific and Technological Development (CNPq, number# 303671/2019–0). Adeliane Castro da Costa and Fábio Muniz de Oliveira received a Ph.D. fellowship from CNPq; Fabrício Camargo received an MSc fellowship from CNPq. Lázaro Moreira Marques‐Neto received a Ph.D. fellowship from CAPES (CAPES, number# 0001). Ana Paula Junqueira‐Kipnis received fellowship from CNPq grants number: 311128/2023–8 and 303671/2019–0. Ana Paula Junqueira‐Kipnis received fellowship from Fundação de Amparo a Pesquisa de Goiás, Centro de Excelência em Tecnologia e Inovação em Saúde (CETI Saúde) : 202410267000884.

## Conflicts of Interest

The authors declare no conflicts of interest.

## Data Availability

The data that support the findings of this study are available from the corresponding author upon reasonable request.
